# Alopecia and hair repigmentation associated with anti-programmed death-ligand 1 (PD-L1) immunotherapy

**DOI:** 10.1016/j.jdcr.2024.09.029

**Published:** 2024-11-19

**Authors:** Jennifer Strong, Vincent I. Poon, Patrick Hallaert, Deneise Francis, Danielle M. Pastor, Leslie Castelo-Soccio, Julius Strauss, Isaac Brownell

**Affiliations:** a Dermatology Branch, National Institute of Arthritis and Musculoskeletal and Skin Diseases (NIAMS) and National Cancer Institute (NCI), National Institutes of Health (NIH), Bethesda, Maryland; b Center for Immuno-Oncology, National Cancer Institute, National Institutes of Health, Bethesda, Maryland; c Office of Research Nursing, National Cancer Institute, Center for Cancer Research, National Institutes of Health, Bethesda, Maryland

**Keywords:** alopecia areata, checkpoint inhibitors, hair repigmentation, immune-related adverse event, immunotherapy

## Introduction

Immune checkpoint inhibitors (ICIs), in particular agents targeting the programmed cell death protein 1/programmed death-ligand 1 (PD-1/PD-L1) axis and cytotoxic T-lymphocyte associated protein 4 (CTLA-4), have become a cornerstone of oncology treatment. These agents are approved for a variety of tumor types and have received tumor-agnostic approval in patients with cancers having a high tumor mutational burden or DNA instability. Although effective in many cases, ICIs are also associated with immune-related adverse events (irAEs), which can manifest in nearly every organ system.

Some of the most common irAE presentations are dermatologic; however, toxicity involving hair is relatively rare. Nonetheless, ICI-related alopecia areata (AA) and universalis^1^ have been reported, as has hair depigmentation, poliosis,^2^ and in 1 case series, hair repigmentation.^3^ The hair follicle is canonically a site of immune privilege, consequently, these events can be thought of as a breaking of that privilege.^2^ We report the occurrence and management of checkpoint inhibitor-associated AA with concurrent global hair repigmentation.

## Case report

A woman in her sixties with metastatic squamous cell carcinoma of the anus including lung and brain metastases was seen by the dermatology consult service for hair loss. Her cancer was previously treated in the localized setting with chemoradiation. Upon progression to metastatic disease, she was treated with palliative cisplatin and 5-fluorouracil. She had subsequent stereotactic radiation to a right lower lobe lung nodule, followed by carboplatin and paclitaxel after disease progression. After progressing on second-line systemic therapy, she was enrolled on 2 sequential clinical trials with immune-modulating agents, both of which included a PD-L1 targeted checkpoint inhibitor. Her course was complicated by grade 2 autoimmune iritis during week 21 of checkpoint inhibitor therapy, which resolved with prednisone eye drops, and by subclinical hypothyroidism, which was managed with thyroid hormone replacement.

**Fig 1:**
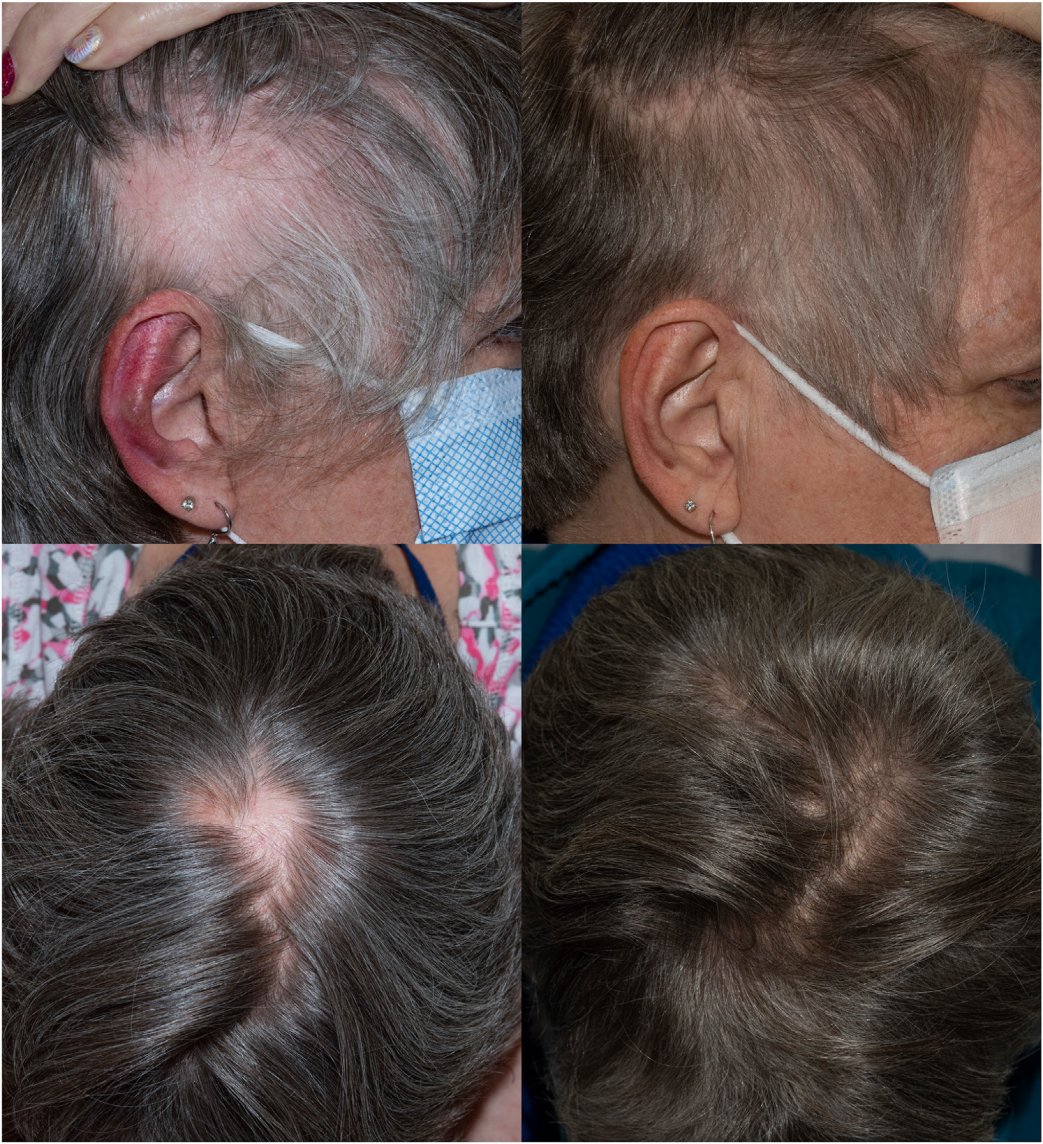
*Left*: discrete round patches of nonscarring hair loss on the right parietal and vertex scalp within a background of age-related hair graying; *Right*: hair regrowth at 6 months in corticosteroid-treated area, also showing generalized repigmentation of scalp hair.

After 60 weeks on combination immunotherapy, the patient began experiencing patches of nonscarring hair loss from the right anterior parietal and left vertex scalp. Exclamation point hairs were noted on trichoscopy. The hair loss progressed to include patches on the midline central scalp, bilateral parietal scalp, and left anterior scalp (Fig 1). The clinical presentation was consistent with ICI-associated AA and tissue sampling was not pursued.

With under 50% of the scalp involved, localized therapy with intralesional steroid injection was used. The patient received a total of 7 injection sessions of 5 mg/mL triamcinolone to the sites of hair loss at 4-week intervals. After 3 injections, there was evidence of hair regrowth in the treated areas, and the patient had full regrowth of her hair after 28 weeks. Interestingly, at baseline, the patient had age-related canities with diffuse mild graying of her scalp hair. During the period of hair regrowth, the patient experienced generalized hair repigmentation, as seen in Fig 1. The patient noted this was a return to her original hair color and no external dyes or pigmented hair products were used. During the following year, the patient’s hair returned to her baseline of diffuse mild graying. At the time of this report, the patient’s hair growth and graying have been maintained for over 18 months without further intervention, and she remains on the clinical trial with stable disease.

## Discussion

Herein, we report a patient with anal squamous cell carcinoma who experienced ICI-related AA and global hair repigmentation. AA and transient global hair repigmentation are both rare irAEs. AA or alopecia universalis has been reported in 1% to 2% of patients on ICIs.^1^ Our patient presented with AA 15 months after the onset of combination immunotherapy, which is delayed relative to the median onset of 5 months reported in 13 previous cases.^2^ In 15 prior cases of hair repigmentation after ICI therapy, the repigmentation was not associated with alopecia.^3^^,^^4^ Our case is unique in that the patient simultaneously experienced 2 rare and likely unrelated irAEs. Sporadic AA is usually associated with no change in color or decreased hair pigmentation restricted to the areas of hair loss. The patient’s hair repigmentation was evenly generalized and not influenced by the areas of alopecia or steroid injections.

Treatment of ICI-related AA is not well characterized.^1^ In prior cases, a variety of treatments have been used, including intralesional triamcinolone and topical clobetasol creams, shampoos, and lotions.^2^ We report full hair regrowth with intralesional triamcinolone while the patient was maintained on immunotherapy. Skin-directed treatment minimized systemic exposure to glucocorticoid therapy for this patient and would not be expected to interfere with her cancer immunotherapy.

In this case, the patient’s AA and hair pigment changes likely originated from a loss of hair follicle immune privilege due to blockade of PD-L1 signaling. Constitutive PD-L1 expression is thought to contribute to hair follicle immune privilege.^5^ Moreover, in cases of ICI-associated diffuse hair loss^6^ and AA,^7^ the inflammatory cells expressed PD-1 and PD-L1. Similarly, the mechanism for ICI-associated hair repigmentation has been proposed to involve autoimmune inflammation caused by disruption of hair follicle immune privilege.^8^ At the same time, the intralesional glucocorticoids administered to the areas affected by AA did not impede repigmentation despite their reversal of the inflammatory hair loss, suggesting a multifactorial immunological mechanism requiring further investigation.

With the increasing use of ICIs, it is important to be aware of irAEs and their treatment in order to avoid discontinuation of immunotherapy. Our case demonstrates that AA and hair repigmentation can occur over a year after the onset of immunotherapy. Considering the psychosocial impact of AA, restoring hair growth with prompt use of intralesional steroids can improve patient quality of life while maintaining patients on their ICI regimen. The observation of another case of transient ICI-related hair repigmentation supports this being a real albeit rare phenomenon; however, the exact etiology remains unclear.

## Conflicts of interest

None disclosed.
